# Research progress of Paris polyphylla in the treatment of digestive tract cancers

**DOI:** 10.1007/s12672-024-00882-9

**Published:** 2024-02-07

**Authors:** Jia Wang, Bao-yi Ni, Jing Wang, Lei Han, Xin Ni, Xin-miao Wang, Lu-chang Cao, Qian-hui Sun, Xin-pu Han, Hu-jun Cui

**Affiliations:** 1 Hongqi Hospital of Mudanjiang Medical University, Mudanjiang, China; 2 Heilongjiang University of Chinese Medicine, Harbin, China; 3 The First Affiliated Hospital of Heilongjiang University of Chinese Medicine, Harbin, China; 4 Guang’anmen Hospital, China Academy of Chinese Medical Sciences, Beijing, China; 5 Graduate College, Beijing University of Chinese Medicine, Chaoyang, China

**Keywords:** Paris polyphylla, Polyphyllin, Digestive tract cancers, Molecular mechanism, Natural medicine

## Abstract

Cancer has become one of the most important causes of human death. In particular, the 5 year survival rate of patients with digestive tract cancer is low. Although chemotherapy drugs have a certain efficacy, they are highly toxic and prone to chemotherapy resistance. With the advancement of antitumor research, many natural drugs have gradually entered basic clinical research. They have low toxicity, few adverse reactions, and play an important synergistic role in the combined targeted therapy of radiotherapy and chemotherapy. A large number of studies have shown that the active components of Paris polyphylla (PPA), a common natural medicinal plant, can play an antitumor role in a variety of digestive tract cancers. In this paper, the main components of PPA such as polyphyllin, C_21_ steroids, sterols, and flavonoids, amongst others, are introduced, and the mechanisms of action and research progress of PPA and its active components in the treatment of various digestive tract cancers are reviewed and summarized. The main components of PPA have been thoroughly explored to provide more detailed references and innovative ideas for the further development and utilization of similar natural antitumor drugs.

## Introduction

In recent years, with the emergence and continuity of poor lifestyles, the digestive tract has become overwhelmed, and the incidence of digestive tract cancers has increased at a high rate. Digestive tract cancers are among the major diseases that threaten human life and health. According to the latest global cancer data released by the World Health Organization International Cancer Research Agency, digestive tract cancers are at the forefront in terms of incidence and mortality. Digestive tract cancers have a wide range of coverage. Esophageal cancer is the most common type of digestive tract cancer. Digestive tract cancers include gastric, liver, gallbladder, pancreatic, colorectal, anal canal cancers. Surgery-centric solutions have become the cornerstone of multiple tumor treatment models. Chemotherapy, another main treatment method for digestive tract cancers, has a good treatment effect; however, it is also accompanied by drug toxicity, which cannot be ignored. With an increase in the chemotherapy cycles, the sensitivity of the tumor cells to chemotherapy drugs decreases and chemotherapy resistance is produced. This can result in tumor recurrence and metastasis, which directly affects treatment efficacy and the long-term survival of the patient [1].

An increasing number of natural products have been found to have antitumor effects, and their mechanisms of action and therapeutic effects have gradually improved through a large number of basic experiments and clinical studies. They have, therefore, become potential therapeutic options in the field of antitumor therapy. Natural drugs have multiple advantages over radiotherapy, chemotherapy, and targeted therapy They are widely available, rich in natural resources, relatively affordable, have low toxicity, have good clinical effects, have unparalleled advantages in the treatment of tumors, and have become a hotspot for the development of more promising antitumor drugs [2].

Paris polyphylla (PPA) Smith Var.yunnnensis (FRANCH.) Hand.-Mazz, is usually called "Chong Lou" in Chinese. The PPA genus originated in an area with a latitude 35° north of East Asia during the Early Oligocene-period. Over the years, with the continuous updates and advancements in science and technology, research on natural medicines, such as PPA, has deepened and developed, The chemical composition, pharmacological effects, and quality control of natural medicines have been widely studied and applied in the fields of medicine and medicinal herbs, especially in many countries in Asia, where there is a much longer history of research and richer clinical experience on these natural medicines..

The main PPA-producing areas are located in Europe, and many are distributed in the East Asian temperate and subtropical regions, especially China. In the past few decades, the effective ingredients of PPA and their potential mechanisms of action have been discovered. Its extensive pharmacological activities include antibacterial, anti-inflammatory, anti-myocardial, antioxidant, organ protection, immunity boosting, and especially significant antitumor activity [3]. In recent years, PPA has been widely used in the clinical treatment of digestive tract cancers by traditional Chinese medicine due to its unique "Jie du" effect and it has achieved good therapeutic effects [4–7]. Therefore, many scholars have begun to study and explore the chemical composition and pharmacological effects of PPA and have found that it can play an inhibitory role in a variety of digestive tract cancers (such as esophageal, stomach, colorectal, and liver cancer) through a variety of mechanisms. Therefore, this review aimed to identify the main active ingredients and pharmacological effects of PPA. We also conducted antitumor evaluations of the individual PPA components to reveal their respective digestive tract cancers prevention and treatment modalities.

## The main active ingredients that contribute to PPA’s antitumor effects

**Fig. 1:**
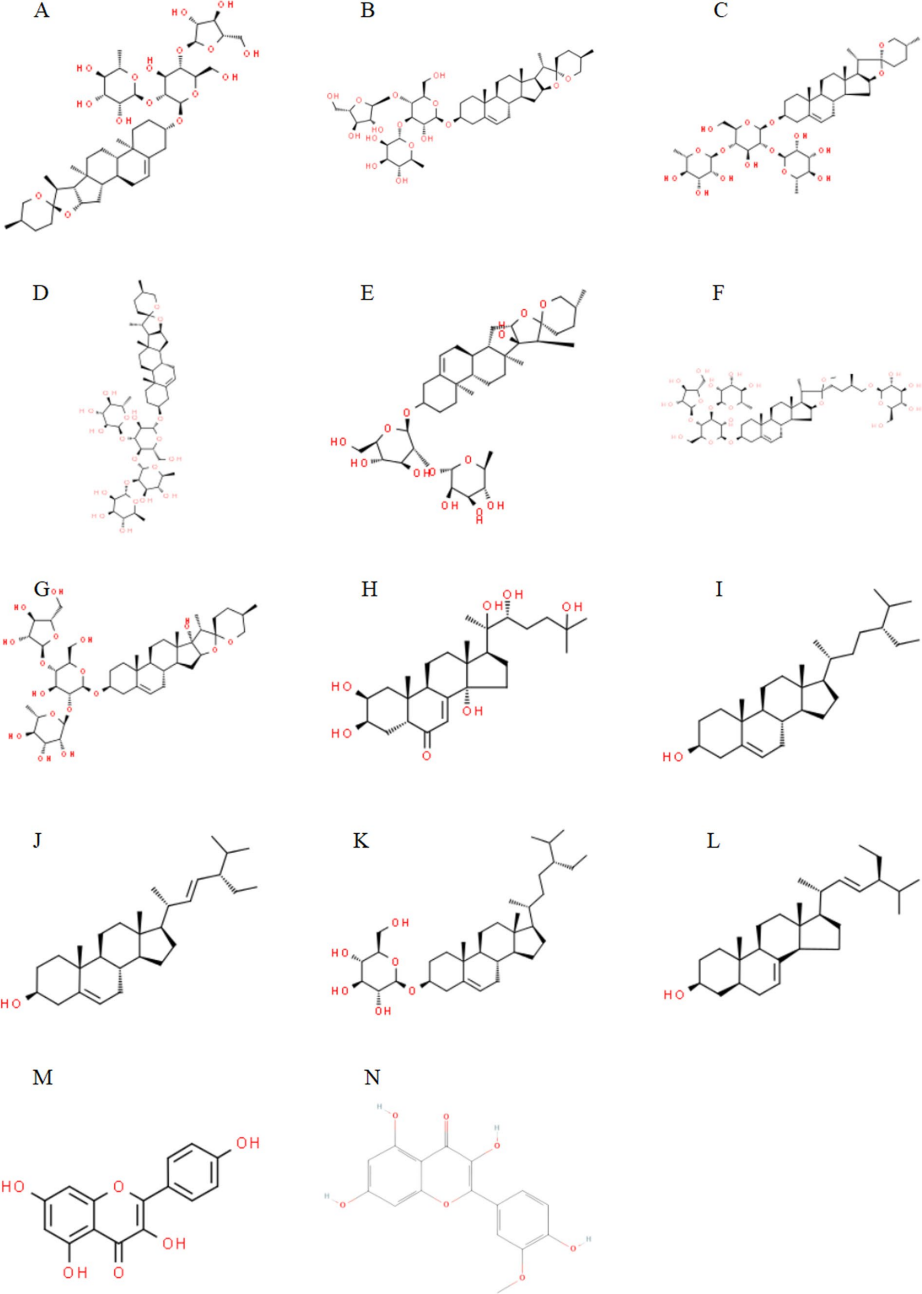
Molecular structureal formula of the main active ingredients of Paris polyphylla. **A** Polyphyllin I (**B**) Polyphyllin II (**C**) Polyphyllin III (Dioscin) (**D**) Polyphyllin V (**E**) Polyphyllin VI (**F**) Polyphyllin VII (**G**) Polyphyllin H (**H**) Beta-ecdysone (**I**) Beta-Sitosterol (**J**) Stigmasterol (**K**) Daucosterol (**L**) Alpha-spinasterol (**M**) Kaempferol (**N**) Isorhamnetin. ((Image credit: National Library of Medicine—National Center for Biotechnology Information https://pubchem.ncbi.nlm.nih.gov/) and ChemSpider Search and share chemistry (http://www.chemspider.com/))

The biologically active ingredients of PPA mainly include polyphyllin (PP), C_21_ steroids, alcohol, flavonoids, trace elements, polysaccharides, and amino acids. PP is the main active ingredient of PPA and is the most important in contributing to PPA’s antitumor effects. In a study on PPA for the treatment of tumors, it was found that digestive tract cancers were more sensitive to PP [8], sterols [9], and flavonoids [10], and that their therapeutic effects were more significant. Figure 1 shows the molecular structures of the main PPA components.

### Polyphyllin

PP is the primary physiologically active and effective ingredient in PPA. According to the glycogen and glycoside contents, PP can be divided into three categories: triteruni saponin, sterite saponin, and alkaline saponin. A total of 142 PP-like components have been isolated [11, 12], most of which are oligosaccharides made from spirostanes combined with sugars, which contain a spiroalkane-or a spirostane-rich skeleton, as well as a cholestane skeleton in their structure. Therefore, researchers have classified steroidal saponins in B. reesei into isospirostanol, spirostanol, furostanol, and pseudospirostanol according to their C25 configuration and the F-ring cyclic state in the spirostanol structure. At present, researchers have isolated more than 90 isospirostanols from the PPA genus, including PP I, II, III, V, VI, VII, A, C, E, F, H, and yanosaponin, etc. [13]. In recent years, an in-depth study on PP, has shown that it has good antitumor effects against a variety of digestive tract cancers. It mainly inhibits cell proliferation, induces apoptosis, regulates cell cycle arrest, induces cell autophagy, and inhibits signaling pathways such as Wnt/β-catenin [14], NF-κB [15], PI3K/Akt/mTOR [16], and MAPK [17], as well and anti-angiogenesis. It can be combined with a variety of chemotherapeutic drugs to significantly effect of improve efficacy and reduce toxicity. In digestive tract cancer, PP has broad developmental prospects as a novel therapeutic and adjuvant drug.

### C_21_ steroids

C_21_ steroids are a class of steroid derivatives containing 21 carbon atoms, of which the glycoside elements are pregnatrienes or their isomers. Recently, researchers have isolated and identified six C_21_ steroidal compounds from the stems and leaves of Dianthus chinensis, including two pregnatriene saponins and four C_22_-steroidal lactone saponins [18, 19].

### Flavonoids

Flavonoids are a class of polyphenolic compounds found in a various plants. The main flavonoids contained in PPA are flavanols, and the glycosides are kaempferol and isorhamnetin. Kaempferol, is an active ingredient in PPA. Its potential in the antitumor field has been described in both in vivo and in vitro studies. Numerous studies have shown that kaempferol can induce apoptosis and cell cycle arrest in cancer cells by regulating several signaling pathways (e.g., PI3K/AKT), stimulating the activation of tumor suppressor genes, inhibiting tumor angiogenesis, and exerting antitumor effects [20]. Isorhamnetin, a flavonoid and another active ingredient of PPA, has received widespread attention because of its anti-inflammatory and anti-proliferative effects in a variety of tumors. It has been shown that isorhamnetin can inhibits pro-inflammatory cytokines and regulates various signaling pathways, such as the MAPK/Akt signaling pathways, to inhibit cell proliferation and cell cycle processes and to exert antitumor effects [21].

### Sterols

PPA contains β-sitosterol, stigmasterol, daucosterol, α-spinasterol, and other plant sterols. Sterols have been found to inhibit the development and progression of many cancers through various cell signaling pathways. β-sitosterol, in addition to its application in the field of antitumor activity, can also be used in mental psychology, it plays a role in relieving mental anxiety, sedation, and analgesia. Stigmasterol is also used for its anti-inflammatory, antioxidant, cholesterol, and cardiovascular disease risk reduction properties, and has potential applications in memory improvement [22]. Daucosterol has antioxidant and neuroprotective effects in addition to its good antitumor effects [23].

### Other components

Other active ingredients of PPA include Paris polyphylla var. chinensis polysaccharide, amino acids, trace elements (e.g., potassium, sodium, and calcium), beta-ecdysone, and fatty acids [24]. In the current study, ti was found that beta-ecdysone was less studied in the digestive tract and exerted antitumor effects in breast cancer cells, such as synergizing with doxorubicin and overcoming multidrug resistance to enhance doxorubicin’s antitumor effects [25, 26].

## The mechanisms behind the antitumor actions of PPA’s active ingredients

### Inhibition of tumor cell proliferation

PP contributes to the main physiological activity of PPA and is the most important component of PPA in exerting its antitumor effects. Some scholars have found that PP I, II, VII, H, etc., which are some of the active ingredients of PPA, can significantly inhibit the proliferation of the tumor cells of esophageal, gastric, hepatic, and colorectal cancers in vitro experiments and play antitumor effects.

In esophageal cancer cells, PPI could inhibit the proliferation of OE-19 cells in a dose-dependent manner [27]. Li et al. found that the PP ethanol extract increased the expression of connexin 26 at the mRNA and protein levels, thus inhibiting the proliferation of esophageal cancer ECA109 cells and exerting antitumor effects [28]. In addition, in gastric cancer cells, He et al. found that PPI significantly inhibited the proliferation of the gastric cancer cells line HGC-27 in a dose-dependent manner (inhibition rate, 3 mg/kg: 78.8% > 1.5 mg/kg: 43.4%), showing better therapeutic effects (inhibition rate, PPI: 78.8% > paclitaxel (PTX): 70.5%) and higher safety (PTX: rapid weight loss) than PTX (10 mg/kg) [29].

Several studies have shown that PPI and II have excellent antitumor cell proliferation effects in hepatocellular carcinoma cells. It was shown that PPI was able to promote hepatocellular carcinoma cell death by activating the mitochondrial pathway [30]. Liao et al. found that PPI was able to inhibit the proliferation of the hepatocellular carcinoma cell lines HepG2 and Huh-7 in a xenograft model [31]. In human hepatocellular carcinoma cells SMCC-7721, PPI was able to inhibit cell proliferation by regulating the PI3K/mTOR signaling pathway [16]; moreover, Pang et al. found that PPII was able to inhibit the proliferation of hepatocellular carcinoma SMCC-7721 cells and exert antitumor effects by inhibiting the AKT/NF-κB signaling pathway [15]. Qin et al. found that PP II inhibited the proliferation of the human hepatoma cell lines HepG2 [32]and BEL7402 [15]. In addition to PPI and PPII, PPH also plays a role in anti-hepatocellular carcinoma cell lines. Chen et al. found that in MHCC-97Hhepatocellular carcinoma cells, PPH was able to regulate the Wnt/β-catenin pathway, inhibit cell proliferation, and exert antitumor effects [14].

In colorectal cancer cells, PP I, II, and VII showed significant antitumor effects. PPI inhibited the proliferation of colon cancer SW480 cells by regulating AKT/mTOR and ROS signaling [8]. Chen et al. found that in colorectal cancer HT-29 and HCT116 cells lines, PPII was able to inhibit the development of colon cancer cells and reduce the size of xenograft tumors by regulating mitochondrial division and the NF-κB signaling pathway [13]. Li et al. found that PPVII could inhibit the proliferation of colon cancer HCT116 cells by downregulating the expression of Wnt pathway factors [33]. Moreover, Li et al. found that PPVII inhibited the proliferation of colon cancer HT-29 and SW-620 cells by regulating the MAPK/AKT signaling pathway [17].

In addition to PP, sterols, another category of the active ingredients of PPA, play an important antitumor role against several cancer species, such as gastric cancer SGC-7901 cells [34], and hepatocellular carcinoma cells. Daucosterol is a sterols, and it has been shown that in hepatocellular carcinoma HepG2 and SMMC-7721 cells, daucosterol was able to inhibit their proliferation in a concentration-dose dependent manner [35].

Kaempferol is a flavonoid that exerts its antitumor effects by regulating the epidermal growth factor receptor (EGFR) signaling pathway and inhibiting the proliferation of KYSE150 and Eca-109 cells in esophageal squamous carcinoma [10]. Kaempferol also inhibits the proliferation of HCT-8/116 colorectal cancer cells by inhibiting TS or attenuating p-Akt activation [36].

Isorhamnetin, is a primary components of PPA. Studies have shown that isorhamnetin can inhibit the proliferation of esophageal cancer Eca-109 cells [37] and promote apoptosis [38] by inhibiting the activation of the NF-κB signaling pathway and COX-2 expression. A tumor-associated hypoxic microenvironment can promote tumor cell proliferation, and hypoxia-induced cellular autophagy is the main mechanism of tumor cell hypoxia protection. It was shown that isorhamnetin was able to inhibit MKN-45 autophagy in gastric cancer cells in a hypoxic environment by targeting PI3K and regulating the PI3K/Akt/mTOR signaling pathway, which inhibits gastric cancer cell proliferation and further development of gastric cancer; therefore isorhamnetin is a promising candidate for the treatment of gastric cancer [39].

### Induction of tumor cell cycle arrest

The ability of cells to complete proliferation is closely related to their ability to move smoothly from one phase of the cell cycle to another. To ensure the orderly progression of the cell cycle, a regulatory process is required for the monitoring and co-coordination of cell cycle checkpoint proteins and a large number of intra- and extracellular signals. If the signal at a certain locus is missing, mutated, damaged, or monitored abnormally, the cell will be stalled at the corresponding stage or checkpoint, will undergo repair of the damage, and will not be able to enter the next stage, a phenomenon known as cell cycle block.The cell cycle block helps to maintain genetic stability and provides extra time for repairing damage repair during cellular damage, thus reducing cellular mutations and playing an important role in suppressing tumor development.

Several studies have shown that PP can inhibits cell growth by inducing cell cycle arrest in a variety of tumor cells and exerting antitumor effects. Yan et al. showed that in hepatoma cells SMMC-7721 cells, PPI, II, III, IV and V can block the cell cycle in the G0/G1 phase and PPVI can block the cell cycle in G2/M phase. In HepG2 cells, PPIV and VI can bolck the cell cycle in the G2/M phase, PP I and III can block the cell cycle in the S phase, and PP II and V increase the proportion of cells in the S and G2/M phases and inhibit tumor growth [40]. Zeng et al. found that in hepatocellular carcinoma cells HepG2 cells, PP I can block cells in the G2/M phase and inhibit tumor cell growth [41]. PPVI induced HepaRG cell cycle blockade in the S phase and inhibited tumor growth by upregulating p21 and downregulating cyclin A2 and CDK2 expression. Thus, it exerts anti-hepatocellular carcinoma effects [42]. Li et al. found that PP-26 can inhibited the proliferation of hepatocellular carcinoma HepG2 cells by triggering a G2/M phase block in a dose-dependent manner [43]. Some studies found that PPII and PPIX exerted cell cycle-blocking effects in colorectal cancer HT-29 and HCT-116 cells. PPII was able to arrest the cell cycle in the G1 phase and inhibit the expression of cyclin D1 and c-Myc in HT-29 and HCT-116 cells [44]; PPIX was able to regulate the PI3K/Akt/GSK3β signaling pathway by regulating p21, CDC2, and CDC25C to block the cell cycle in the G2/M phase [45].

In colorectal cancer HCT116 cells, PPI was able to regulate cell cycle proteins, such as p21 and cyclin B1 expression, and trigger excessive ROS, which induced HCT116 cells to block in the G2/M phase and exert antitumor effects [46]. In gastric cancer cells, PPI inhibited the development of gastric cancer by promoting the conversion of LC3-I to LC3-II and down-regulating cyclin B1, which induces cell cycle arrest in the G2/M phase in the gastric cancer cell line HGC-27 [29].

Downregulation of the proapoptotic gene p53 is closely associated with the development of many tumors. Zhong et al. found that β-sitosterol induce apoptosis, S-phase cell cycle blockade, and exerted antitumor effects in AGS gastric cancer cells by activating the p53 signaling pathway and upregulating pro-apoptotic genes in vitro [9]. Qu et al. found that stigmasterol could induce apoptosis in liver cancer cells and block the G1/G0 phase cycle in colorectal cancer by regulating the expression of the p53/PI3K/Akt signaling pathway and exerting antitumor effects [47].

Kaempferol is a flavonoid, that exerts antitumor effects in vitro and in vivo by participating in cell cycle regulation. It regulates cyclin expression to block the cell cycle of KYSE150 and Eca109 cells in the G0/G1 phase, therefore kaempferol is be a clinically effective candidate for the treatment of esophageal squamous carcinoma [10].

### Induced apoptosis and autophagy of tumor cells

Apoptosis and autophagy are both molecular mechanisms by which cells maintain the stability of their organelles and internal environment. There is a complex interaction between apoptosis and autophagy, as they are co-activated by various emergency stimuli, share multiple regulatory molecules, and even coordinate each other’s transformation. Apoptosis is an active process and refers to the ability of the cell to maintain the stability of the internal environment; it is initiated by the cell itself and results in its own autonomous and orderly death, and this process is strictly co-regulated by a variety of genes. Many factors induce and activate apoptosis, including oxidative damage, mitochondrial damage, radiation, and drug release. Tumor generation and development may be directly or indirectly related to the disturbance in the apoptotic process.

Several studies have shown that several PP can induce apoptosis and inhibit tumor progression by upregulating pro-apoptotic genes. Li et al. found that PP ethanol extract can increased the expression of connexin 26 at the mRNA and protein levels, which induced apoptosis in esophageal cancer ECA109 cells and exerted antitumor effects [28]. In the gastric cancer cells line SGC7901/DDP, PPI could induces apoptosis by regulating CIP2A/PP2A/AKT pathway gene expression [48]. Zheng et al. found that PPI inhibited the growth of gastric cancer MKN-45 and AGS cells by promoting the nuclear apoptosis of cancer cells [49]. In addition, PPI was able to reduced Ki-67 expression in xenograft tumors in the HGC-27 gastric cancer cell line and increased the percentage of apoptotic cells to exert antitumor effects [29].

In HepG2 hepatocellular carcinoma, PPI, II, and V can significantly induce apoptosis and exert antitumor effects [40]. PPI can induce apoptosis through intracellular and extracellular apoptotic pathways [41]. PPVII can induce apoptosis in HepG2 cells through MAPK and PTEN/p53 pathways [50]. Li et al. found that PP-26 can inhibit Akt/ GSK-3β/Foxo3 signaling pathway expression, while downregulating caspase-3, and-9, PARP, Bcl-2, and MCL-1, and upregulating BAX expression to induce apoptosis in hepatocellular carcinoma HepG2 cells [43]. In hepatocellular carcinoma cells HepG2 and MHCC-97H cells, PPVI increased ROS levels and thus, induced apoptosis [51]. PPVI induces apoptosis in HepaRG cells by promoting ROS-mediated mitochondrial dysfunction [42]. Yan et al. found that in hepatocellular carcinoma SMMC-7721 cells, PPI, V, and VI significantly inducted apoptosis [40]; and Luo et al. found that PPI could induce apoptosis in colon cancer SW480 cells by promoting ROS production and regulating the AKT/mTOR pathway [52]. In colon cancer HT-29 and HCT-116 cells, PPII was able to induce apoptosis and inhibit cell colony formation to exert antitumor effects [44]; PPIX was able to induce apoptosis by regulating the PI3K/Akt/GSK-3β signaling pathway, upregulating Caspase-3 and-9 and BAX and downregulating B-cell lymphoma 2 (Bcl-2) [45]. Moreover, PPVII regulated the RAS signaling pathway to induce apoptosis in colon cancer HT-29 and SW-620 cells and inhibit their malignant progression of colon cancer cells [17]. In addition, another PP extract can exerted antitumor effects by increasing ROS and activating caspase-3, thereby, inhibiting HCT-116 cell metastasis and inducing apoptosis [53].

Sterols, as one of the active ingredients in PPA, also play a role in the induction of apoptosis. Stigmasterol induces apoptosis in gastric cancer MGC-803 cells by up-regulating pro-apoptotic genes in a dose-dependent manner, thus effectively inhibiting cell proliferation and suppressing tumor progression dose [54]. Kim et al. showed that stigmasterol can induce apoptosis in HepG2 cells by upregulating the expression of pro-apoptotic genes (caspase-8 and-9, BAX and p53), downregulating the expression of the anti-apoptotic gene Bcl-2, and damaging the cellular DNA; thus, it has great potential for application in the treatment of hepatocellular carcinoma treatment [55]. Mitochondrial damage is also an important pathway that causes apoptosis, and stigmasterol activates the mitochondrial apoptotic pathway and induces apoptosis in gallbladder cancer cells by regulating p27 and JAB1 expression [56].

Matrix metalloproteinases (MMPs) cleave the extracellular matrix and can induce apoptosis. It was shown that in human colon cancer HCT116/HT29 cells, kaempferol intervention resulted in decreased tumor cell viability, decreased cell migration rates, many transmembrane cells, increased early apoptosis, increased number of G1 phase cells, decreased number of S phase cells, and significantly downregulated expression of MMP1, -2 and -9. This suggests that kaempferol significantly inhibits proliferation, invasion, and migration of tumor cells and induces apoptosis, thereby exerting antitumor effects [57]. Li et al. showed that kaempferol induces apoptosis in HCT-8/116 colorectal cancer cells by upregulating BAX and downregulating Bcl-2 and TS expression levels [36].

In patients with pancreatic cancer, high TGM2 expression was positively associated with a poor prognosis.Kaempferol induces ROS-dependent apoptosis by downregulating TGM2 and the Akt/mTOR signaling pathway in PANC-1 and Mia PaCa-2 pancreatic cancer cells. By modulating TGM2, kaempferol may exert additional antitumor effects that need to be explored further [58]. Kaempferol has been shown to induce apoptosis in esophageal squamous carcinoma Eca-109 cells through the upregulation of caspase-3 and-9, BAX and the downregulation of Bcl-2 expression in a concentration-dependent manner to exert antitumor effects [59].

In gastric cancer AGS-1 and HGC-27 cells, isorhamnetin can upregulate BAX/Bcl-2 expression, leading to decreased mitochondrial potential and ROS accumulation. This indicates that isorhamnetin can induce apoptosis in gastric cancer cells through the mitochondrial apoptotic pathway, which infers its great therapeutic potential in the treatment of gastric cancer treatment [60]. Gallbladder cancer is the most common biliary tract tumor with a poor prognosis, and Isorhamnetin has been shown to induce apoptosis and block the cell cycle in the G2/M phase in gallbladder cells by regulating the PI3K/Akt signaling pathway [61].

Autophagy is the process by which cells utilize lysosomal mechanisms to degrade and reuse damaged organelles, misfolded proteins, and other macromolecules. This is a routine step in cell growth, development, and for the maintenance of homeostasis in the internal environment. Autophagy plays an important role in both preventing and promoting cancer development. Autophagy can promote cancer development by promoting starvation or autophagy and degrading apoptotic tumor cells, thus enabling continued survival of the cancer cells. The current theory suggests that autophagy is both a tumor suppressor and a positive factor in tumor cell survival. However, a growing body of research evidence suggests that autophagy is likely to be used as a cancer suppressor. Therefore, promoting selective targeting of autophagy to inhibit tumor formation and progression may be an important direction to take for effective cancer prevention and treatment in the future.

Luo et al. found that PP I promoted autophagic death in colon cancer SW480 cells by promoting ROS production and regulating the AKT/mTOR pathway [52]. PPI also exerted antitumor effects by reducing cell viability and inducing ROS-dependent autophagy in HCT116 cells [46]. Lin et al. found that in colorectal cancer DLD-1 cells, the PP ethanol extract induced cell death by upregulating autophagic markers without triggering p53- and caspase-3-dependent apoptosis [62]. In gastric cancer cells, PPI inhibited the autophagy-regulated PDK1/Akt/mTOR signaling pathway in vivo and in vitro, and induceed cellular autophagy in the gastric cancer cells line HGC-27 [29]. Zhang et al. found that PPVII induced autophagic cell death by activating the JNK pathway and inhibiting the PI3K/AKT/mTOR pathway in hepatocellular carcinoma HepG2 cells [63]. Components other than PP, such as kaempferol, can induce autophagy in gastric cancer cells by activating the IRE1/JNK/CHOP signaling pathway [64].

### Inhibition of tumor invasion and metastasis

Tumor cells that have invaded and metastasized share the same malignant behavior and ability as the original tumor cells and have important links to tumor progression. Invasion refers to aggressive malignant tumor cells crossing the extracellular matrix layer or basement membrane stroma layer and invading from the primary region to another region, while metastasis refers to tumors entering the body's circulatory system (e.g., blood or lymphatic system) from the primary site and spreading through the circulatory system to other organs or tissues far away from the primary site to recolonize in the new environment [65]. Tumor metastasis is an important cause of tumor recurrence and is a key factor in cancer-related deaths.

Mitochondria are important participants in apoptosis, and mitochondrial dynamics (mitochondrial fission and fusion) are key factors for mitochondria to perform normal physiological functions, and dysfunction in these dynamics is closely related to the occurrence of diseases. Mitochondrial dynamics ensures normal mitochondrial function, maintenance of normal mitochondrial morphology, and play an important role in apoptosis or apoptosis-related diseases through dynamic regulation of mitochondrial morphology. Mitochondrial division [66] and autophagy [67] often occur during apoptosis, and mitochondrial fusion promotes cell survival. Lin et al. found that PP I and VI can inhibit mitochondrial fusion and cell invasion in colon cancer Caco-2 cells [68].

Angiogenesis, the process of blood vessel formation, which provides essential nutrients and oxygen to tumor cells, is critical for tumor invasion and metastasis, and therefore, anti-angiogenesis research is also extremely important. PPD was found to significantly inhibit the migration and capillogenesis of microvascular endothelial HMEC-1 cells and showed significant angiogenic defects, indicating the potential of PPD in anti-angiogenic studies [69]. Some scholars have found that in cholangiocarcinoma xenograft tumors, stigmasterol can significantly reduce the expression of tumor necrosis factor-alpha (TNF-α) and inhibit further tumor angiogenesis after intervention, thus inhibiting tumor progression [70].

Epithelial-mesenchymal transition (EMT) is an important step in cancer development and metastasis that confers tumor cells with the ability to metastasize and invade cells, including stem cell characteristics, reduction in apoptosis and senescence, and promotion of immunosuppression. EMT promotes the development of an aggressive phenotype during cancer metastasis [71]. It has been shown that TGF-β1 promotes EMT-induced metastasis and inhibits apoptosis in many tumor types; the deletion of the expression of E-cadherin, an inducible gene that inhibits EMT, is thought to be a critical step in causing EMT and cancer metastasis. Zhang et al. found that PPI inhibited TGF-β1-induced invasiveness of gastric cancer SGC790 cells and upregulated E-cadherin expression in them; PPI could also partially inhibit the expression of the CIP2A/PP2A/AKT pathway, which inhibited the invasive deterioration process of gastric cancer cells [48].

Cancer stem cells (CSCs) have stem cell properties, such as self-replication and multicellular differentiation, which can give rise to tumors through self-renewal and differentiation into multiple stem cell types. These cells are hypothesized to persist in tumors as distinct populations and to generate new tumors, leading to tumor recurrence and metastasis [31]. Therefore, the development of specific therapies targeting CSCs is expected to improve the survival and quality of life of cancer patients, especially those with metastatic diseases. Zhu et al. found that CD44 was highly expressed in colon cancer tissues, and PPVII could inhibit the sphere-forming ability of colon cancer cells by down-regulating CD44 expression, which inhibited the development of colon CSC [72]. In addition to the above, PPI can inhibit HCT116 cell invasion and metastasis by regulating E-cadherin expression [73]. Kaempferol can inhibit the invasion and metastasis of hepatocellular carcinoma Huh-7 and SK-Hep-1 cells by inhibiting the MMP-9 and Akt signaling pathways, further proving the anticancer effects and mechanisms of kaempferol [74]. In hepatocellular carcinoma HepG2 and SMMC-7721 cells, daucosterol inhibits their migration and invasion by regulating the Wnt/β-catenin signaling pathway in a concentration-dependent manner [35].

## Combination of radiotherapy and drug resistance reversal

Chemotherapy is one of the most important therapies for tumors and has significant therapeutic effects, but the generation of multidrug resistance and chemotherapy side effects are important bottlenecks in enhancing its therapeutic effects. Therefore, the development of effective complementary therapies can help improve the therapeutic effects of chemotherapy on tumors and control its side effects. In recent years, to effectively alleviate chemotherapy resistance and its side effects, the research on complementary therapies and adjuvant measures has become more in-depth and is gradually being applied clinically. Some studies have shown that PPA and its active ingredients, have significant clinical efficacy in the fields of combined radiotherapy, chemotherapy, and relief of drug resistance. Basic experimental results and possible mechanisms of action of PPA and its active ingredients are briefly described.

Several studies have shown that PP can be combined with chemotherapeutic agents to achieve chemo-sensitization. Song et al. found that the IC_50_ value of cisplatin was reduced when combined with PPI to treat gastric cancer SGC-7901 cells, and that this combination promoted cisplatin-induced G2/M phase cell cycle arrest and apoptosis, suggesting that PPI is a potential sensitizer for cisplatin treatment of gastric cancer [75]. Li et al. found that PP-26 synergized with 5-FU to enhance its inhibitory and antitumor effects on the proliferation of hepatocellular carcinoma HepG2 cells [43]. In colorectal cancer DLD-1 cells, the PP ethanol extract could enhanced the cytotoxic, therapeutic and antitomor effects of Adriamycin on tumor cells [62]. The PP extract can be combined with 5-FU/cisplatin to enhance its colorectal cancer therapeutic significance [53].

The breast cancer resistance protein (BCRP/ABCG2) was first identified in multidrug-resistant breast cancer cells. High expression of BCRP/ABCG2 can affect the absorption, distribution, and metabolism of tumor drugs, and can even result in excretion of tumor drugs, directly leading to drug resistance. BCRP/ABCG2 expression is associated with a poor prognosis in various human tumors. β-sitosterol, an active ingredient of PPA, inhibits BCRP/ABCG2 to reverse the drug-resistance of drug-resistant colorectal cancer cells to oxaliplatin [76].

Constitutive activation of the aromatic hydrocarbon receptor (AhR) is an important cause of cisplatin resistance in esophageal squamous carcinoma cell lines with significant upregulation of the drug resistance protein ABCG2. It has been shown that kaempferol intervention significantly inhibits the upregulation of ABCG2, reverses ABCG2-mediated multidrug resistance, and acts as an AhR antagonist; this provides new data to support the idea that natural compounds in combination with chemotherapeutic agents can achieve a reduction in chemo-sensitization and toxicity reduction [77].

Aerobic glycolysis is associated with tumor growth and resistance to chemotherapy. Kaempferol has been shown to inhibit the glycolytic process by promoting miR-326 expression, regulating the miR-326-hnRNPA1/A2/PTBP1-PKM2 signaling axis, decreasing glycoconjugate and lactate formation, and ultimately reversing 5-FU resistance in colon cancer HCT8-R cells [78]. It can also enhance 5-FU resistance in colorectal cancer HCT-8/116 cells by regulating the PI3K/Akt signaling pathway [36]. Therefore, kaempferol plays an important role in overcoming 5-FU resistance in cancer cells. Another study showed that kaempferol combined with oxaliplatin significantly inhibited tumor cell proliferation, enhanced oxaliplatin’s anticancer effects, and reduced oxaliplatin chemoresistance in colorectal cancer HCT116 and HT29 cells [79].

Erlotinib(ERL) is an EGFR tyrosine kinase inhibitor (TKI) used to treat pancreatic cancer. The clinical efficacy of ERL is limited by the activation of bypassing EGFR signaling. Network pharmacology results suggest that kaempferol-sensitizes pancreatic cancer cells to ERL treatment, and that this effcet may be associated with the PI3K/AKT signaling pathway and EGFR-TKI resistance. Kaempferol treatment significantly downregulated the expression levels of p-EGFR, p-AKT, p-ERK1/2, and Bcl-2, while kaempferol in combination with ERL significantly inhibited pancreatic cancer cell proliferation and induced apoptosis through upregulation expression of caspase-9, PARP, and BAX. These results suggest that kaempferol can be used as a chemosensitizer to enhance the therapeutic effects of ERL and reduce its toxicit by inhibiting the PI3K/Akt and EGFR signaling pathways, and that it may be a clinical candidate for the effective treatment of pancreatic cancer [80].

In hepatocellular carcinoma cells, the combination of kaempferol and doxorubicin showed stronger inhibition and better clinical therapeutic potential in terms of proliferation, cell cycle progression, DNA damage, mitochondrial function, migration, and invasion of hepatocellular carcinoma cells [81]. ABT-199 (Venetoclax), the first selective Bcl-2 inhibitor, is ineffective against hepatocellular carcinoma cells when used alone. When kaempferol is combined with ABT-199, it induces apoptosis by downregulating the expression of the anti-apoptotic proteins Bcl-2, Bcl-xL, and MCL-1 and upregulating BAX expression [82]. Sorafenib is the only FDA-approved drug that is routinely used for the treatment of advanced hepatocellular carcinoma. Despite the remarkable effecacy of sorafenib in the treatment of hepatocellular carcinoma, multidrug resistance remains its biggest problem. Studies have shown that Kaempferol can achieve synergistic anticancer effects by reducing P-glycoprotein overexpression, reversing MDR, and enhancing the sensitivity of hepatocellular carcinoma HepG2 and N1S1 cells to sorafenib [83]. These results suggest that kaempferol combined with chemotherapy has potential applications in the treatment of hepatocellular carcinoma.

Several studies have shown that isorhamnetin can inhibit Nrf2 expression, activate PPAR-γ and cause cellular autophagy through regulating the MAPK/Akt pathway to suppress hepatocellular carcinoma cell proliferation, metabolism, and EMT; Additionally, isorhamnetin has potential therapeutic value by inhibiting TNF-α in response to patients with sorafenib-resistant hepatocellular carcinoma; isorhamnetin can also antagonize TGF-β1 to enhance the efficacy of dosorubicin and reduce its toxic side effects [21]. Manu et al. showed that the therapeutic effect of isorhamnetin combined with capecitabine was superior to capecitabine alone, and could significantly inhibit the viability of tumor cells, the expression of tumor markers, inhibit the activation of the NF-κB signaling pathway, and enhance the antitumor effects of capecitabine [84].

Radiotherapy is also one of the basic means of tumor treatment, but tumor radiation resistance remains a major therapeutic obstacle. It has been found that rhizoma paridis total saponins can enhance radiotherapy sensitivity by being able to downregulate the MUC-1 protein [85].

## Clinical research

PPA is a traditional Chinese antitumor drug and one of the ingredients of the Ganfule, Boling, Lou Lian capsules, and other Chinese patented antitumor drugs, which are widely used and recognized for their efficacy in clinical practice [86]. The Xing Jian Decoction is an effective prescription for the clinical treatment of gastric cancer after surgery, and PPIII is one of the main components of this drug. Compared with the control group, which only received chemotherapy, the clinical symptoms and quality of life of the patients in the combination group who received the traditional Chinese medicine were significantly improved, and the EORTC QLQ-STO52 scale and FACT-G scores were statistically significant [87]. The Yiqi Huayu Jiedu Decoction is an effective prescription for the clinical treatment of liver cancer in traditional Chinese medicine, and PP I is one of the effective components of this drug that plays an anticancer role. Clinical studies have shown that the 6 month, 1 year and 2 year survival rates of patients treated with sorafenib combined with sorafenib are significantly higher than those of patients in the control group treated with sorafenib alone (95.2% VS 72.7%, 85.7% VS 50.0%, and 47.6% VS 13.6%, respectively). PFS in the combination group was significantly higher than that in the control group (6.99 ± 0.60 months VS 4.55 ± 0.41 months) [88].

## New technologies / novel drug delivery systems

The extraction of highly active ingredients from PPA for disease prevention and treatment is an important direction to follow for the development of new drugs. However, because PPA-active ingredients vary and have various pharmacological properties, their solubility, stability, and oral absorption have not been completely resolved, and the low bioavailability of these compounds is one of the main limitations of their disease controlling effects.

Novel nanoparticle-based formulations can overcome these limitations and improve the problem. Wang et al. developed a PPI self-microemulsifying drug delivery system (PPI-SMEDDS), and evaluated the quality and in vivo pharmacokinetics of PPI. The results of the in vitro experiments showed that the cumulative release rate of the PPI-SMEDDS reached 80% within 2 h. The in vitro antitumor effect was significantly better than that of PPI, which lays the foundation for further research on new PP dosage forms [89]. β-sitosterol has poor water solubility, low bioavailability, and a short elimination half-life, which greatly limits its therapeutic applications. Andima et al. optimized and surface modified β-sitosterol and encapsulated it into nanoparticles, the modified β-sitosterol showed significant cytotoxic potential and good anticancer activity, and the tumor cell viability was inhibited up to 80%; therefore, encapsulating β-sitosterol nanoparticles would be a promising strategy [90]. Kaempferol is predominantly found in various fruits and plants. Studies by Mohammad Imranet al. have shown that kaempferol can be used in the form of chitosan nanoparticles, gold nanoclusters, and submicron emulsions, which allow it to achieve higher bioavailability and exert more pronounced inhibitory effects in tumor models. The study also showed that kaempferol can be used in the form of chitosan nanoparticles, gold nanoclusters, and submicron emulsions [91]. Carboxymethyl chitosan, which is carboxylated from chitosan, has good water solubility and biodegradability and is one of the best choices as a nanomaterial. Isorhamnetin is an important anticancer drug. Yang B et al. showed in their study that the use of Carboxymethyl chitosan as a nanocarrier and modification of the particle surface with polydopamine resulted in the formation of Isorhamnetin/Carboxymethyl chitosan-polydopamine nanorods, which exerted the effect of targeting tumor cells and growth inhibition [92].

## Prevention of precancerous lesions

The occurrence and development of cancer is a multifactorial, multi-stage process with slow evolution. Benign lesions in some organs of the human body are prone to the heterogeneous proliferation of cells, and tend to undergo malignant changes that are referred to precancerous lesions. Pre-cancerous lesions are the intermediate stage of the transition from normal benign lesions to malignant lesions, and they are more likely to become malignant tumors under the action of many carcinogenic factors. Some precancerous lesions are reversible, and the treatment of precancerous lesions is a key stage in tumor prevention and the best time for treatment. Common pre-cancerous lesions in the digestive tract include mild atypical hyperplasia of the esophagus and gastrointestinal tract, chronic atrophic gastritis, gastric ulcers, chronic hepatitis, liver cirrhosis, polyps, and colitis.

Stigmasterol significantly inhibited colon shortening, decreased fecal heme content, and lowered colitis pathology scores in vivo in mice. It also significantly reduced the mRNA expression of the inflammatory factors IL-1β, IL-6, MCP-1, and COX-2 in the colon tissue. Beta-sitosterol also significantly reduced the colitis pathology score but significantly inhibited the expression of IL-6 and COX-2 [93].

Alcoholic liver disease, liver fibrosis, and nonalcoholic steatohepatitis are important contributors to hepatocellular carcinoma. Daucosterol, a phytosterol with hepatoprotective properties, is a components of PPA. There are many causes of liver injury, including liver failure and alcoholic liver disease. Studies have shown that daucosterol intervention can effectively reduce liver injury, reduce liver index, TNF-α and IL-1β/6 expression levels, and attenuate liver injury in mouse models of liver failure by regulating the IL-6/STAT3 signaling pathway [94]. Daucosterol also plays a key role in the prevention of liver cancer by improving alcohol-induced liver injury through the p38/NF-κB/NLRP3 signaling pathway, as well as preventing liver oxidative stress and lipid accumulation [95]. Liver fibrosis is an important factor leading to cirrhosis and subsequently liver cancer, and the activation of hepatic stellate cells is the main cause of liver fibrosis. Studies have shown that isorhamnetin inhibits the TGF-β/Smad signaling pathway, blocks ROS production, reduces oxidative stress, inhibits hepatic stellate cell activation, and prevents the development of liver fibrosis [96].

High concentrations of ROS can activate inflammatory signaling pathways, such as the MAPK/JAK-STAT/Wnt, pathways leading to different degrees of cellular degradation and liver injury, creating an inflammatory environment, and contributing to the development of liver disease [97]. The inflammatory environment leads to the increased expression of pro-fibrotic and pro-inflammatory genes, which promote the formation of a tumor microenvironment and ultimately lead to the development of hepatocellular carcinoma. Kaempferol has been shown to exert antioxidant and anti-apoptotic effects by modulating signaling pathways, such as the AMPK/PTEN/PI3K-Akt pathways, reducing inflammatory responses, protecting hepatocytes, exerting inflammation prevention, inhibiting tumor growth, and has therapeutic potential for the prevention of precancerous lesions [98, 99]. Daucosterol was able to significantly attenuate dextran sulfate sodium-induced colitis and prevent the development of colorectal cancer by reducing ROS production, macrophage infiltration, expression of pro-inflammatory factors such as TNF-α and IL-6/1β, and increasing NK cell activity [100].

There is a strong link between the bile acid-gut microbiota axis and the risk of colorectal cancer. Kaempferol was shown to reverse the decreasing trend of goose deoxycholic acid and 12α-hydroxylated BAs by upregulating the expression of sterol 27-hydroxylase, sterol 12α-hydroxylase, and FXR, effectively regulating the bile acid system and gut microbiota, reducing tumor load and restoring the intestinal barrier. This is effective for the signficant prevention of precancerous lesions in colorectal cancer is significant [101].

## Discussion

Digestive tract cancer is a major category of malignant tumors with poor prognoses. The symptoms of cancer and the adverse reactions and toxicity caused by anti-cancer treatments seriously affect human health and quality of life. As a traditional natural product, PPA has a long history of use and has good clinical effects. Several studies have suggested the significant efficacy of PPA against digestive tract cancers. However, PPA, an active ingredient of Chinese herbal medicines, is complex, and its specific anti-cancer mechanisms and its clinical applications need to be further summarized and explored. This study reviewed the research results and clinical applications of PPA and its active components in digestive tract cancers.

In this paper, the main active ingredients, possible mechanisms of action, and combined applications with modern medicine of PPA were reviewed. Details are presented in Table 1. And the Composition-Target table, which is detailed in Table 2.

**Table 1:** Tumor types and characteristics of various active components of Paris polyphylla

Type	Composition	Tumor type	Synergistic reaction	Features
Polyphyllin and Their Metabolites	I, II, III, IV, V, VI, VII, IX, 26, H	Esophageal, Stomach, Liver, and Colon	Cisplatin, Doxorubicin, and 5-FU	①Wide range of antitumor effects; ②Antitumor effects are exerted through multiple pathways; ③Synergistic effects can be produced in combination with multiple antitumor therapies to improve efficacy and reduce toxic side effects; ④Combined with novel drug delivery systems; ⑤Prevention of precancerous lesions
Flavonoid-O-glycoside	Kaempferol, Isorhamnetin	Esophageal, Stomach, Liver, Colon, Pancreas, and Gallbladder	Cisplatin, Doxorubicin, Oxaliplatin, Erlotinib, ABT-199, Sorafenib, Capecitabine, and 5-FU	
Sterols	β-Sitosterl, Stigmasterol, Daucosterol	Stomach, Liver, Colorectal, and Gallbladder	Cisplatin, Gemcitabine, Oxaliplatin, and Paclitaxel

**Table 2:** Active Ingredients and Targets/ Pathways of Paris polyphylla

Active ingredient	Target	Signal pathway	Phenotype	Cancer species
Polyphyllin I	P21, cyclinB1, E-cadherin, LC3-II, p62, p-GSK-3β, p-p70S6K, p-PDK1, p-Akt, p-mTOR	PI3K/mTOR, Akt/mTOR, ROS, CIP2A/PP2A/AKT,PDK1/Akt/mTOR,Akt/GSK-3β/β-catenin	Proliferation, Cycle arrest, Apoptosis/Autophagy, Invasion, Combined chemotherapy,	Esophagus, Stomach, Liver, Colorectum
Polyphyllin II	MMP2/9, p-AKT, p-NF-κB, CyclinD1, c-Myc	Akt/NF-κB	Proliferation, Cycle arrest, Invasion	Liver, Colorectum
Polyphyllin III	–	–	Cycle arrest	Liver
Polyphyllin V	–	–	Cycle arrest	Liver
Polyphyllin VI	p21, cyclin A2, CDK2	ROS	Cycle arrest, Invasion	Liver, Colorectum
Polyphyllin VII	caspase-3, p-MEK1/2, p-ERK1/2, p-AKT, p-GSK-3β	MAPK/Akt, PTEN/p53, Ras, JNK, PI3K/AKT/mTOR	Proliferation, Apoptosis/Autophagy	Colorectum, Liver
Polyphyllin IX	p21, cdc2, cdc25C, caspase3, 9, Bax, Bcl-2	PI3K/Akt/GSK3β	Cycle arrest, Apoptosis	Liver
Polyphyllin H	β-catenin	Wnt/β-catenin	Proliferation	Liver
Rhizoma paridis total saponin	MUC-1	–	Combined radiotherapy	Liver
Polyphyllin 26	caspase3, 9, PARP, Bcl-2, Mcl-1, Bax	Akt/GSK-3β/Foxo3	Cycle arrest, Proliferation, Apoptosis, Combined chemotherapy	Liver
Polyphyllin Ethanol extract	connexin26, Bcl-2, Bad, LC3, Beclin-1	–	Proliferation, Apoptosis, Combined chemotherapy	Esophagus, Colorectum
Kaempferol	TGM2, FXR, casapase-3/9, Bcl-xL, TS, ABCG2, Mcl-1, Bax, Bcl-2, MMP-1/2/9, P-glycoprotein, CYP27A1, CYP8B1,	Akt/mTOR, ROS, IRE1/JNK/CHOP, EGFR, miR-326-hnRNPA1/A2/PTBP1-PKM2, AMPK/PTEN/PI3K/Akt	Proliferation, Cycle arrest, Apoptosis/Autophagy, Combined chemotherapy, Prevention of precancerous lesions	Esophagus, Colorectum, Pancreas, Stomach, Liver, Colorectum
Isorhamnetin	COX-2, Bax, Bcl-2, Nrf2, PPAR-γ, TGF-β1	NF-κB, PI3K/Akt/mTOR, ROS, MAPK/Akt, TGF-β/Smad	Proliferation, Cycle arrest, Apoptosis, Combined chemotherapy, Prevention of precancerous lesions	Esophagus, Stomach, Cholecyst, Liver
Daucosterol	TNF-α, IL-1β/6	Wnt/β-catenin, IL-6/STAT3, p38/NF-κB/NLRP3, ROS	Invasion, Prevention of precancerous lesions	Liver, Colorectum
β-sitosterol	P53, IL-6, BCRP/ABCG2, COX-2	P53, AMPK/PTEN/Hsp90	Cycle arrest, Apoptosis, Combined chemotherapy, Prevention of precancerous lesions	Stomach, Colorectum
Stigmasterol	P27, jab1, IL-1β/6, MCP-1, p53, COX-2, caspase-8/9, Bax, Bcl-2	p53/PI3K/Akt	Cycle arrest, Apoptosis, Prevention of precancerous lesions	Liver, Cholecyst

We summarized the anti-digestive tract cancer effects of various active ingredients of PPA and found that PP and its compounds were the most important active ingredients with anti-digestive tract cancer effects.

**Fig. 2:**
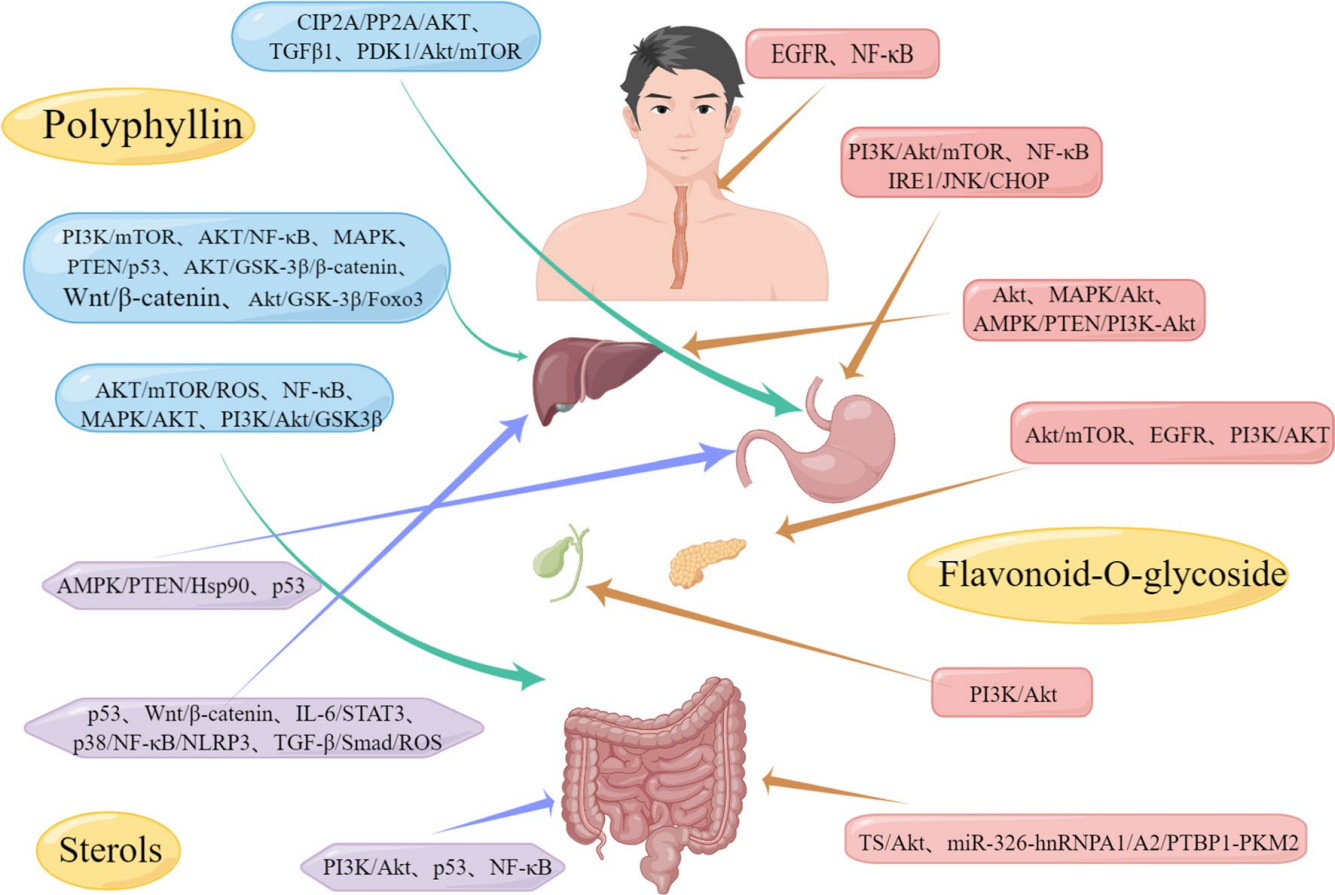
Molecular mechanism/pathway of the antitumor effects of Paris polyphylla (Image credit: By Figdraw (https://www.figdraw.com)

Therefore, we conducted an in-depth investigation into the mechanisms and clinical applications of various chemical monomers of PP against digestive tract cancers. The results showed that PP acted on multiple processes of growth and differentiation of various digestive tract cancer cells, including inhibition of cell proliferation, induction of cell apoptosis, blocking of the cell cycle, inhibition of cell invasion and metastasis, regulation of autophagy, and reversal of drug resistance in tumor cells. The common biological features of most malignant tumors include uncontrolled proliferation, invasion, and metastasis; however, active ingredients such as PP can achieve targeted regulation of these signaling pathways by activating or inhibiting certain key genes, thus playing an antidigestive role, which shows the potential of PPA in antidigestive tumors. Further details are shown in Fig. 2.

A large amount of in vitro experimental data have proven that PPA has definite antitumor effects; however, there are some flaws and shortcomings. Due to the incomplete development system of PPA monomer reagents, data from in vivo experiments and clinical trials are relatively scarce and lack real-world data support. There is a lack of relevant studies based on systems biology and integrated pharmacology, and the pharmacokinetics of the in vivo metabolic process of PPA and its active ingredients still need to be further explored. In recent years, studies on the antitumor effects of PPA have mainly focused on the secondary metabolites of the dried roots and rhizomes of PPA; however, there are few reports on the pharmacological effects of other components of PPA, which also provides new research ideas for later researchers.

Therefore, in view of the current research status, it is urgent to improve the following aspects in future in-depth research and development: (1) Continue to explore the mechanisms behind the antitumor effects of PPA in depth, and at the same time accelerate the development and clinical trials of PPA monomer reagents, realize the mutual corroboration of in vivo and in vitro experiments, and improve the clinical and real-world data support; (2) Currently, research on PPA focuses on cytotoxicity and less on immunotherapy, which can be further studied in terms of activating and promoting the transformation of macrophages, dendritic cells, and helper T cells; (3) Natural drugs often have poor water solubility, low bioavailability, and therefore, other problems, and should be structurally modified and computer-aided design using advanced drug delivery technology, carrier technology and nanotechnology in modern pharmacy, so that other active ingredients, such as sterols and flavonoids, can be explored and researched in greater depth; (4) Conduct more in-depth research on the toxicology of PPA and its active ingredients, control the dosage relationship between its efficacy and toxicity, and increase the safety of clinical medications; (5) The development of modern molecular biology, cell biology, bioinformatics, systems biology and other disciplines will help to elucidate and deepen the studies on the antitumor mechanisms of action of PPA, which should be oriented to clinical application so as to make an organic combination of advanced technology and natural medicines.

## Conclusion

With abundant resources, moderate prices, and low toxicity, natural drugs have unparalleled advantages in the treatment of cancer and have become a hot spot in the development of more promising antitumor drugs. PPA is a natural medicine, and its chemical composition and pharmacological action are mainly based on PP. It has broad applicational prospects and developmental potential as an adjuvant drug for the treatment of digestive tract cancers. In terms of its clinical applications, PPA promotes the therapeutic effects of radiotherapy, chemotherapy, and targeted therapy, and greatly reduces the toxic side effects and adverse reactions associated with antitumor therapy. However, there are still some shortcomings in the current research. It is hoped that more effective ingredients can be found in natural medicines in the future to provide more effective treatment plans and options for the clinical treatment of digestive tract cancers.

## Data Availability

Not applicable.
